# Pharmacokinetics and tissue distribution of monotropein and deacetyl asperulosidic acid after oral administration of extracts from *Morinda officinalis* root in rats

**DOI:** 10.1186/s12906-018-2351-1

**Published:** 2018-10-24

**Authors:** Yi Shen, Qi Zhang, Yan-bin Wu, Yu-qiong He, Ting Han, Jian-hua Zhang, Liang Zhao, Hsien-yeh Hsu, Hong-tao Song, Bing Lin, Hai-liang Xin, Yun-peng Qi, Qiao-yan Zhang

**Affiliations:** 1 School of Pharmacy, Fujian University of Traditional Chinese Medicine, No. 1 Qiuyang Road, Shangjie Town, Minhou County, Fuzhou, 350122 People’s Republic of China; 2 School of Pharmacy, Zhejiang University of Traditional Chinese Medicine, Gaoke Road, Fuyang District, Hangzhou, 310053 People’s Republic of China; 3 School of Pharmacy, Second Military Medical University, No. 325 Guohe Road, Yangpu District, Shanghai, 200433 People’s Republic of China; 4 Department of Pharmacy, Eastern Hepatobiliary Surgery Hospital, Second Military Medical University, No. 225 Changhai Road, Yangpu District, Shanghai, 200438 People’s Republic of China; 5 Department of Biotechnology and Laboratory Science in Medicine, National Yang-Ming University, No. 155, Section 2, Li Nong Street, Beitou District, Taipei, 112-21 People’s Republic of China; 6 Fuzhou General Hospital of Nanjing Military Region, No. 156, West Second Ring North Road, Gulou District, Fuzhou, 350025 People’s Republic of China

**Keywords:** *Morinda officinalis*, Iridoid glycosides, Pharmacokinetics, Tissue distribution, UPLC-MS/MS

## Abstract

**Background:**

Iridoid glycosides (IGs), including monotropein (MON) and deacetyl asperulosidic acid (DA) as the main ingredients, are the major chemical components in *Morinda officinalis* How. (MO) root, possessing various pharmacological properties including anti-osteoporosis, anti-inflammation and anti-rheumatism activities.The aim of the present study was to further elucidate the pharmacological actions of MO by investigating the pharmacokinetics and tissue distribution of IGs in MO.

**Methods:**

An ultra high performance liquid chromatography-tandem mass spectrometry (UHPLC-MS) method was developed and validated for simultaneous determination of MON and DA levels in plasma and various tissues of Wistar rats. MON, DA and acetaminophen (ACE) as the internal standard (IS) were extracted from rat plasma and tissue samples by direct deproteinization with methanol. The rats were administered orally at 1650 mg/kg MO and 25, 50 and 100 mg/kg MO iridoid glycosides (MOIGs) or intravenously at MOIG 25 mg/kg for pharmacokinetic study of MON and DA. In addition, 100 mg/kg MOIG was administered orally for tissue distribution study of MON and DA. Non-compartmental pharmacokinetic profiles were constructed. Tissue distributions were calculated according to the validated methods.

**Results:**

Significant differences in the pharmacokinetic parameters were observed in male and female rats. The AUC_0-t_, C_max_ and bioavailability of MON and DA in female rats were higher than those in male rats. MON and DA mainly distributed in the intestine and stomach after oral administration, and noteworthily high concentrations of MON and DA were detected in the rat hypothalamus.

**Conclusion:**

The results of the present study may shed new lights on the biological behavior of MOIGs in vivo*,* help explain their pharmacological actions, and provide experimental clues for rational clinical use of these IGs extracted from the MO root.

**Electronic supplementary material:**

The online version of this article (10.1186/s12906-018-2351-1) contains supplementary material, which is available to authorized users.

## Background

The root of *Morinda officinalis* How (MO), also named as “Bajitian” in traditional Chinese medicine [1], has long been used as a tonic or nutrient supplement to prevent and treat multiple diseases including osteoporosis, depression, rheumatoid arthritis, impotence and Alzheimer disease in China, South Korea, Japan and Southeast Asia [2–6]. These pharmacological properties are believed to be mainly attributed to oligosaccharides, polysaccharides, iridoid glucosides, antharaquinines and volatile oil as the main chemical constituents in the MO root [7–9]. Monotropein (MON) and deacetyl asperulosidic acid (DA), the two major MO iridoid glycosides (MOIGs), accounts for more than 2% in the root of MO. Previous investigations showed that MON possessed anti-nociceptive, anti-inflammatory and anti-osteoporotic activities [3, 8, 10–12]. For example, MON could protect against chondrocyte apoptosis and catabolism induced by Interleukin 1β (IL-1β), and improve inflammatory medium of RAW 264.7 macrophages and dextran sulfate sodium (DSS)-induced colitis via the NF-κB pathway [11, 12]. In our previous work on chemical compounds of the MO root [13], we extracted MOIGs from the MO root by using an optimal technical method and found that content of MON and DA was greater than 60%, suggesting potential therapeutic applications of MOIGs in the treatment of inflammatory and bone diseases such as osteoarthritis, rheumatoid arthritis and inflammatory bone loss.

Some recent studies [14, 15] used a new LC-MS/MS method to determine the plasma concentrations of MON and DA for pharmacokinetic study in rats. However, there are scant studies to describe the absorption properties, tissue distribution and oral bioavailability of MON and DA in vivo, and there is little knowledge about the pharmacokinetics and target organs/tissues of these two compounds. The primary goal of this study is to clarify the in vivo distribution and action mechanism of the two compounds by analyzing the pharmacokinetics and tissue distribution. Firstly, MON and DA levels in the plasma and tissue of Wistar rats were simultaneously detected by using ultra high performance liquid chromatography-tandem mass spectrometry (UHPLC-MS), knowing that it is a simple, rapid and reliable assay. Secondly, the pharmacokinetic, tissue distribution and bioavailability of MON and DA were determined in both sexes of Wistar rats after oral and intravenous administration of MOIG and MO ethanol extracts, hoping that the results could provide useful information for the research and development of IGs extracted from the MO root.

## Methods

### Chemicals, reagents and animals

**Fig. 1:**
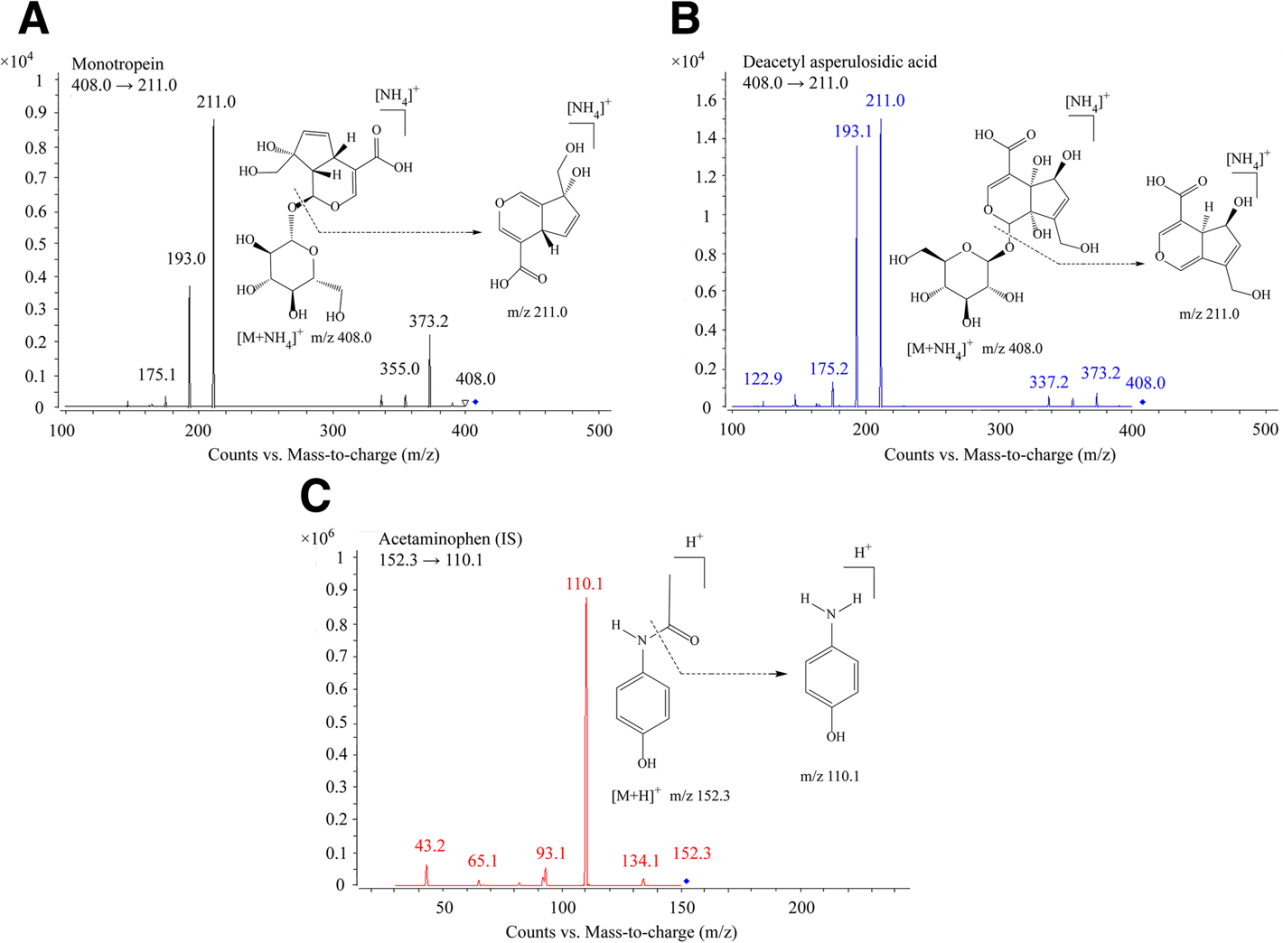
Product ions and structures of MON (**a**), DA (**b**), and IS (**c**)

MON and DA were purchased from Shanghai Yuanye Biological Technology Co., Ltd. The purity was more than 98%, and their chemical structures were verified by NMR, MS and HPLC. ACE (C_8_H_9_NO_2_, purity > 98%) purchased from the National Institute for the Control of Pharmaceutical and Biological Products (Beijing, China) was used as internal standard (IS). The chemical structures for MON, DA and ACE are shown in Fig. 1. HPLC grade acetonitrile, methanol and formic acid were obtained from Merck Company (Darmstadt, Germany). All the other reagents were of analytical-grade purity, and purchased from Sinopharm Chemical Reagent Co. Ltd. Deionized water was generated by a Milli-Q system from Millipore (Milford, MA, USA).

The MO root was collected from Zhangzhou of Fujian Province of China in October 2017, and identified by Professor Qiao-yan Zhang of the Department of Pharmacognosy, the Second Military Medical University School of Pharmacy (Shanghai, China). The voucher specimen (MO 20171008) was deposited in the herbarium of this Department. MOIG and MO ethanol extracts were prepared in our laboratory. Briefly, 2.0 kg powder MO root was extracted under permeation with 32.0 L solution of ethanol-water (70:30, *v*/v) for 20 h, and then filtrated. The combined filtrate was concentrated under reduced pressure to obtain the MO extract. Then, the MO extract was diluted with water to obtain 1.0 g crud drug /mL working solution. The MO extract (1.0 g crud drugs /mL) was adsorbed to XDA-1 macroporous adsorption resin, and eluted with water and 10% ethanol. The 10% ethanol elute collected was centrifuged to obtain MOIGs. The yield of MOIGs was 2.4%, and the content of MON and DA in MOIGs was 38.6% and 23.6%, respectively. The content of MON and DA in the MO extract was 1.27% and 0.65%, respectively.

Thirty-six male and 36 female healthy Wistar rats (200-220 g) aged 8 weeks were purchased from Sippur Will Kay Company and housed at the Experimental Animal Center of the Second Military Medical University (Certificate No. SCXK 2013–0016). The rats were acclimatized for a week on a 12 h light-dark cycle under a temperature of 24 ± 0.5 °C and humidity of 47.5 ± 2.5% before drug administration. All animals were fasted for 12 h before initiation of the experiment, with free access to water during the course of the experiment.

### UHPLC-MS/MS equipment and method

An Agilent series 1290 UHPLC system (Agilent, USA) was used in this study. An Agilent ACE3C_18_-PFP column (3.0 × 150 mm, 3.0 μm) was used as the stationary phase and the column temperature was maintained at 35 °C. The mobile phase (A) was methanol containing 0.1% formic acid and 5 mM ammonium formate, and mobile phase (B) was water containing 0.1% formic acid and 5 mM ammonium formate. The program of gradient elution was as follows: 6% B phase at 0–2 min, 6–60% B at 2–3 min, 60–60% B at 3–6 min with a flow rate of 0.4 mL/min. The auto-sampler was conditioned at 4 °C and the injection volume was 1 μL.

The MS detector was composed of an Agilent 6470 tandem mass spectrometer (Agilent technologies, USA) combined with an Agilent Jet Stream Technology (AJS) electrospray source interface (ESI). The mass spectrometric detection was optimized in the positive ion detection mode by multiple reaction monitoring (MRM). The main working parameters of the mass spectrometer are summarized as follows: capillary 4000 V, nebulizer 40 p.s.i., drying gas 10 L/min, gas temperature 350 °C and fragmentor 110 V for analyte and IS, sheath gas temperature 350 °C, sheath gas flow 11 L/min, collosion energy 10 eV for analyte and 20 eV for IS, fragmentation transitions were m/z 408 → 211 for analyte and m/z 152.3 → 110.1 for IS. Data acquisition and analysis were performed using Agilent Mass Hunter Work Station version B.07.00.

### Preparation of calibration standards and quality control samples

Stock solutions (1.0 mg/mL) of MON and DA were prepared in water. The two standard stock solutions were mixed at a high concentration. The mixed solutions were diluted with methanol to obtain the final linearity concentrations of 2–5000 ng/mL for MON and DA, respectively. The IS stock solution (1.0 mg/mL) was also prepared in methanol and diluted to a final working concentration of 10 ng/mL. Quality control (QC) samples were also prepared similarly at the concentrations of 5, 1000 and 4000 ng/mL. All working solutions were stored at 4 °C before use.

### Sample preparation

The plasma or tissue homogenate was thawed to room temperature 25 °C. 50 μL plasma or tissue homogenate with 100 μL IS solution (10 ng/mL) and 50 μL methanol were added into an 1.5 mL eppendorf tube, and mixed using vortex for 30 s. The mixture was centrifuged (11,000×g) at 4 °C for 10 min. Then, 100 μL of the supernatant was transferred into the sample bottle and 1 μL of the supernatant was injected into the UHPLC-MS/MS system for analysis.

### Method validation

Linearity, sensitivity, specificity, accuracy, precision, recovery, matrix effect and stability of the method were validated under the guidelines set by the United States of Food and Drug Administration (FDA) [16] and European Medicines Agency (EMA) [17].

### Specificity and sensitivity

The specificity of the method was evaluated by comparing the chromatograms of blank samples (plasma/tissue homogenate) with blank samples spiked with MON, DA, and real samples after oral administration of the MO extract. Endogenous interference was identified by analyzing six individual blank samples.

### Linearity of calibration curves and LLOQ

Calibration curve samples were prepared in triplicate as previously described and analyzed. The linear curve was generated by plotting the peak area versus the theoretical concentrations of the calibration standard. The lower limit of quantification (LLOQ) was defined with a signal-to-noise ratio of 10:1 with precision and accuracy below 20%.

### Accuracy and precision

Three QC samples on the same day (*n* = 5) were detected to evaluate the intra-day precision and accuracy. The inter-day precision and accuracy along with the standard calibration curve (*n* = 15) were determined at the same procedure for 3 consecutive days. The intra-day and inter-day precisions were evaluated by RSD, and the accuracy was evaluated by RE. The accuracy (RE%) and precision (RSD%) should be within ±15%.

### Extraction recovery and matrix effect

The extraction recoveries of MON and DA were estimated by comparing the observed peak areas of the prepared QC samples with those of non-processed samples at six replicates. The matrix effect (ME) was evaluated by comparing the peak areas of the post-extracted blank plasma/tissue homogenate samples with those of analytes from neat standard samples at three different QC concentrations.

### Stability

The stability was evaluated by the RE of analysis in plasma samples at three levels of QC during storage and handling conditions: three freeze/thaw cycles, 6 h stability at room temperature, auto-sampler for 24 h and storage at − 80 °C for 30 days. The accuracy (RE) should be below 15%.

### Carry-over and dilution

The upper limit of quantification (ULOQ) was detected by the double-blank sample in order to evaluate carry-over. And the peak area of the double-blank sample should be less than 15% of LLOQ, while the IS should be lower than 5%.

Dilution integrity was evaluated by diluting the samples with the 10-fold concentration of ULOQ to 10, 20 and 40 μg/mL for the standard plasma by blank samples matrix (dilution has already covered the concentration range of actual samples). And five parallel processing samples of every dilution level were verified. Finally, the accuracy (RE) and precision (RSD) should be below 15%.

### Pharmacokinetic study

All animal treatments in this study were approved by the Administrative Committee of Experimental Animal Care and Use of the Second Military Medical University in accordance with the National Institute of Health guidelines on the ethical use of animals. According to the results of the previous pharmacodynamic experimental study, 30 rats of both sexes were equally randomized to five groups and orally administered with 25, 50 and 100 mg/kg MOIG and 1650 mg/kg MO extract (the quantities of MON and DA are equal to those in 50 mg/kg MOIG) and administered intravenously with 25 mg/kg MOIG, all of which were dissolved in physiological saline for administration. 0.4 mL heparinized plasma samples were collected from the ophthalmic venous plexus with a sterile capillary tube at pre-administration (time = 0) and oral post-administration (time = 0.167, 0.333, 0.5, 0.75, 1, 1.5, 2, 2.5, 3, 5, 7, 10 and 24 h), and intravenous post-administration (time = 0.033, 0.083, 0.167, 0.333, 0.5, 0.75, 1, 2, 3, 5, 7, 10, 12 and 24 h), respectively. At the end of the experiment, all rats were sacrificed by cervical dislocation. The samples were timely centrifuged at 11,000×g at 4 °C for 10 min, and then 100 μL aliquot of supernatant plasma was transferred into another tube and stored at − 20 °C until analysis.

### Tissue distribution study

Forty-two rats of both sexes were randomized to seven groups and orally administered with 100 mg/kg MOIG. The rats were sacrificed by cervical dislocation, and various kinds of tissue samples, including the small intestine, large intestine, stomach, spleen, ovary, uterus, heart, kidney, marrow, liver, lung, thymus, hypothalamus and testis were collected at 0.5, 1, 2, 4, 8, 12 and 24 h (6 rats at each time point) and washed with normal saline solution, blotted on filter paper, and then weighed. The tissue samples were homogenized with 10 times of the normal saline solution (*w*/*v*). Then the homogenates were centrifuged at 11,000×g at 4 °C for 10 min, then 1.0 mL aliquot of supernatant homogenates was transferred into another tube and stored at − 20 °C until analysis.

### Data analysis

The pharmacokinetic parameters, including area under the plasma concentration-time curve (AUC_0-t_), the area under the plasma concentration-time curve from zero to time infinity (AUC_0-∞_), mean residence time (MRT), half-life (t_1/2_), peak time (T_max_), peak concentration (C_max_), body clearance (CL), and apparent volume of body distribution (V_d_) were calculated using PK Solver 2.0 of Microsoft Excel under the non-compartmental model. The absolute oral bioavailability (F_oral_) of MON and DA from MOIG and MO extracts after oral administration was calculated using the following formula:

F_oral_ = (AUC_oral_ · Dose_i. v._)/(AUC_i. v._ · Dose_oral_) × 100 % .

## Results

### Method development

Knowing that it is very important to efficiently eliminate protein and potential interferences in bio-samples before UPLC-MS/MS analysis, the effects of acetonitrile, acetonitrile-methanol (1:1, *v*/v) and methanol were evaluated on protein elimination. Finally, methanol was found to be superior to the other solutions and therefore used as the precipitation reagent. The matrix effect was between 85 and 110% for the bio-samples treated with methanol precipitation.

MRM is very powerful for pharmacokinetic study due to high sensitivity, selectivity and specificity. In this work, the predominant ions of MON ([M + NH_4_]^+^), DA ([M + NH_4_]^+^) and IS ([M + H]^+^) in the Q1 spectrum were used as the precursor ion to obtain the product ion spectra. The most sensitive mass transitions were m/z 408 → 211 for MON and DA, and m/z 152.3 → 110.1 for IS (Fig. 1). The working parameters of MS/MS were optimized to maximize the analyte response. Under these conditions, the retention time was 3.7 min (MON), 4.2 min (DA), and 5.2 min (IS) in the real samples, and no endogenous interference was observed in the real samples**.**

### Method validation

#### Specificity

**Fig. 2:**
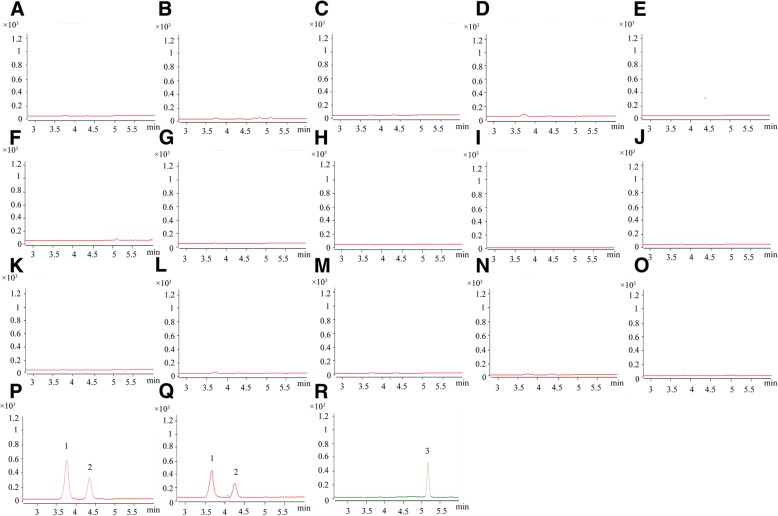
Representative chromatograms of analytes from rat plasma and tissue. Blank plasma and tissue sample: (**a**) plasma, (**b**) liver, (**c**) stomach, (**d**) kidney, (**e**) uterus, (**f**) heart, (**g**) small intestine, (**h**) large intestine, (**i**) spleen, (**j**) lung, (**k**) thymus, (**l**) hypothalamus, (**m**) ovary, (**n**) testis, and (**o**) marrow. (**p**) actual sample plasma at 2 h after orally administration of 100 mg/kg MOIG, (**q**) hypothalamus sample obtained at 2 h after oral administration of 100 mg/kg MOIG, and (**r**) was IS (10 ng/mL). Peak 1 was MON and peak 2 was DA, and peak 3 was acetaminophen (IS)

The specificity of the method was determined by comparing the typical chromatograms with UPLC-MS of blank plasma/tissue homogenates, black samples spiked with MON, DA and IS. The actual plasma samples after oral administration of MOIGs are shown in Fig. 2. The retention time of MON, DA and IS was 3.7, 4.2 and 5.2 min, respectively. Due to the high specificity of MRM mode, no significant endogenous interference was observed.

### Calibration curve and LLOQ

A linear regression was used to evaluated the linearity by the 1/concentration (1/X) weighting analysis in the given concentration ranges of 2–5000 ng/mL for MON and DA in plasma and tissue samples. The calibration curves, coefficients and linear ranges of MON and DA in plasma and each tissue are listed in Additional file 1: Table S1. The calibration curves for all matrices showed good linearity (*R* >0.99) over the concentration ranges. The LLOQs of MON and DA were both 2 ng/mL for plasma and tissue samples, with accuracy less than 20.0% and the precision within ±15%.

### Precision and accuracy

As shown in Additional file 2: Table S2, the intra-day accuracy ranged from − 9.14% to 4.28% for MON and from − 7.72% to 0.46% for DA, while the intra-day and inter-day precision were within 9.26% for MON, and 5.75% for DA, demonstrating that the assay precision and accuracy of the analysis were within the acceptable range.

### Extraction recovery and matrix effect

As presented in Additional file 3: Table S3, the matrix effect of MON, DA and IS was 85.87–109.26%, illustrating no significant ion inhibition or enhancement in this method. The extraction recoveries ranged between 62.11–107.42% for MON, DA and IS, which were also acceptable.

### Carry-over and dilution

No residue was detected in this infinity UHPLC-MS/MS method. The results of the integrity dilution experiment are shown in Additional file 4: Table S4, indicating that the precision was under 10% and the accuracy was within ±15%, which were also acceptable.

### Stability

The results of the stability test are shown in Additional file 5: Table S5, indicating that MON and DA were stable in the plasma at indoor temperature for 6 h, at 4 °C in the auto-sampler for 24 h, after three free-thaw cycles, they were kept at − 80 °C for 30 days.

### Pharmacokinetic study

**Fig. 3:**
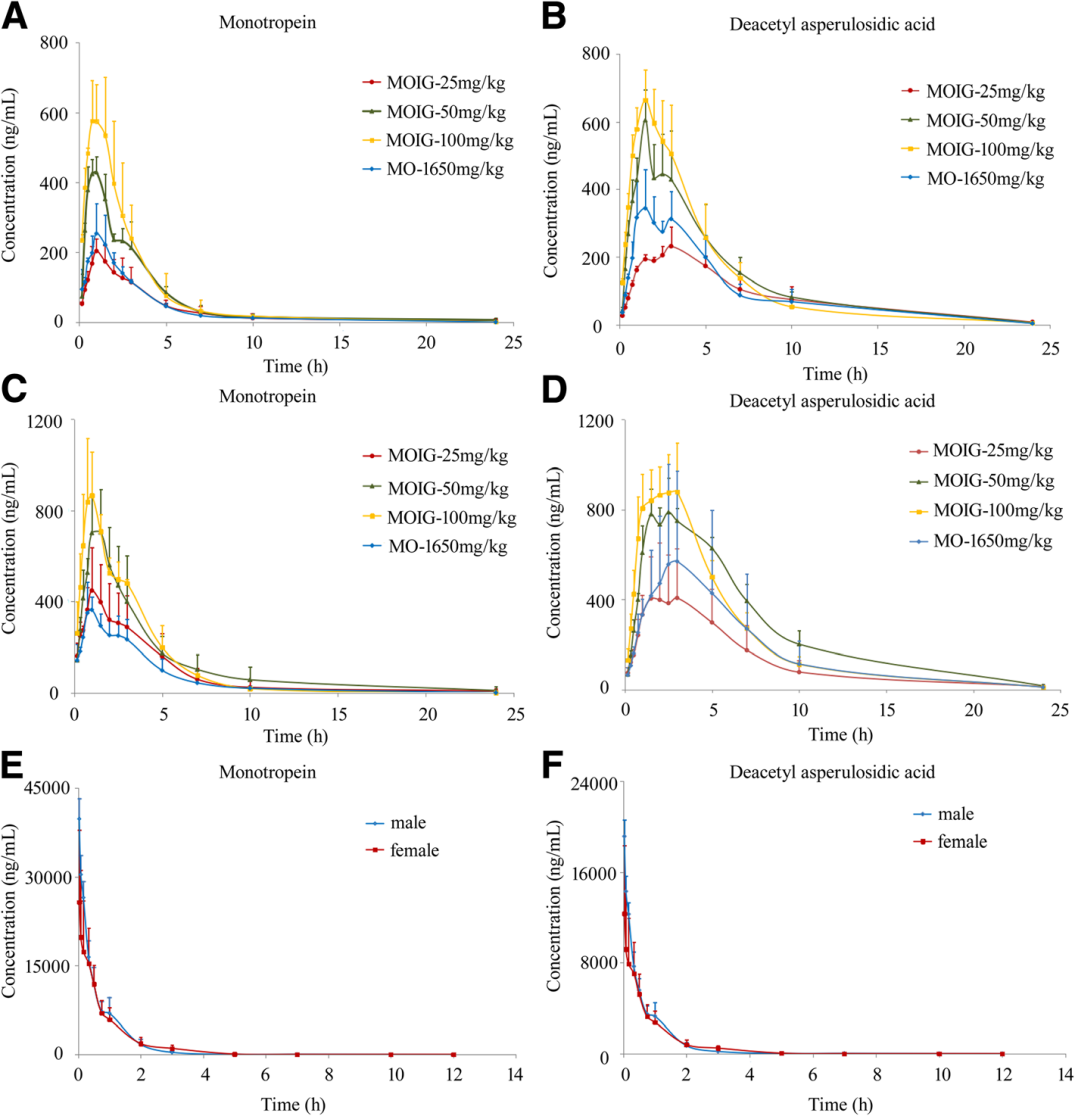
The mean plasma concentration-time profiles of MON and DA after oral administration of the MOIG at a dose of 25, 50 and 100 mg/kg and the MO extract of 1650 mg/kg in male rats (**a** and **b**), female rats (**c** and **d**) or intravenous administration of 25 mg/kg MOIG (**e** and **f**) in rats (*n* = 3, mean ± SD)

The mean plasma concentration-time curves are displayed in Fig. 3. The primary pharmacokinetic parameters are enumerated in Tables 1 and 2. The time from intravenous administration at a dose of 25 mg/kg MOIG to reaching the maximum concentration (T_max_) for both MON and DA was 0.03 h in both male and female rats; the maximum plasma concentration (C_max_) of MON and DA was 39,748 ± 3398 μg/mL and 19,126 ± 1461 μg/mL in male rats, and 25,719 ± 12,174 μg/mL and 12,340 ± 5992 μg/mL in female rats, respectively. MON and DA were shown to have a low apparent volume of distribution (V_d_ from 0.003 ± 0.002 L/g to 0.011 ± 0.004 L/g), with a half-life time (t_1/2_) from 1.76 ± 1.32 h to 2.20 ± 1.86 h and a clearance from 0.001 ± 0.000 to 0.003 ± 0.001 L/(g·h). After oral administration of 3-dose levels of MOIGs, C_max_ versus the MON and DA dose distribution was linear with a correlation coefficient being more than 0.90. The increase in C_max_ of MON and DA was positively correlated with the increase in MOIG dosage. The T_max_ of MON and DA were observed about 1 h and 2 h after oral administration respectively, demonstrating that the blood circulatory system could absorb MON and DA. The value of T_max_ and t_1/2_ demonstrated that MON and DA were relatively slowly dispersed. Apparent volume of distribution (V_d_) indicated that MON and DA were taken up in the tissue after oral administration, with an absolute bioavailability value of 2.04–3.69% and 8.29–16.12% for MON in male and female rats, and 3.90–10.66% and 16.17–37.23% for DA in male and female rats at an oral dose of 25, 50 and 100 mg/kg MOIG, respectively. These results indicate that the bioavailability of these drugs was dose dependent and showed a significant gender difference.

**Table 1:** The main pharmacokinetic parameters after oral administration of 25, 50, and 100 mg/kg for MOIG, and 1650 mg/kg for MO or intravenous administration of 25 mg/kg MOIG in male rats (*n* = 3, mean ± SD)

Parameters	Component	po-MOIG-25	po-MOIG-50	po-MOIG-100	po-MO-1650	iv-MOIG-25
AUC_0-t_ (μg·h/L)	MON	832 ± 322	1505 ± 189	1777 ± 537	875 ± 182	21,501 ± 4877
	DA	2010 ± 545	3262 ± 423	3366 ± 753	2220 ± 820	10,239 ± 2044
AUC_0-∞_ (μg·h/L)	MON	872 ± 334	1585 ± 279	1812 ± 550	877 ± 182	21,500 ± 4859
	DA	2069 ± 557	3301 ± 411	3397 ± 748	2286 ± 773	10,239 ± 2044
AUMC_0-∞_ (μg·h/L)	MON	5121 ± 2698	8645 ± 5804	5011 ± 2132	3064 ± 882	15,255 ± 5098
	DA	14,739 ± 5191	17,844 ± 2090	15,476 ± 3663	12,867 ± 5938	8079 ± 2340
MRT (h)	MON	5.71 ± 1.96	5.17 ± 2.58	2.68 ± 0.40	3.47 ± 0.43	0.70 ± 0.09
	DA	7.03 ± 0.68	5.41 ± 0.24	4.58 ± 0.15	5.43 ± 0.94	0.78 ± 0.09
t_1/2_ (h)	MON	4.60 ± 1.41	7.11 ± 2.18	2.76 ± 1.84	3.00 ± 0.45	1.85 ± 1.67
	DA	4.80 ± 0.62	3.80 ± 1.01	3.76 ± 0.64	3.65 ± 1.16	2.90 ± 0.65
T_max_ (h)	MON	1.00 ± 0.00	0.92 ± 0.14	0.92 ± 0.52	1.17 ± 0.29	0.03 ± 0.00
	DA	2.33 ± 0.76	2.00 ± 0.87	1.5 ± 0.00	2.17 ± 0.76	0.03 ± 0.00
V_d_ (L/g)	MON	0.20 ± 0.04	0.32 ± 0.04	0.22 ± 0.13	8.51 ± 2.61	0.004 ± 0.004
	DA	0.09 ± 0.04	0.08 ± 0.03	0.17 ± 0.06	3.90 ± 0.90	0.011 ± 0.004
CL (L/g·h)	MON	0.031 ± 0.010	0.032 ± 0.006	0.059 ± 0.020	1.932 ± 0.361	0.001 ± 0.000
	DA	0.013 ± 0.004	0.015 ± 0.002	0.030 ± 0.006	0.791 ± 0.310	0.003 ± 0.001
C_max_ (μg/L)	MON	203 ± 34	451 ± 35	605 ± 114	265 ± 82	39,748 ± 3398
	DA	236 ± 54	624 ± 63	664 ± 89	379 ± 91	19,126 ± 1461
F (%)	MON	4.06 ± 1.55	3.69 ± 0.65	2.11 ± 0.64	2.04 ± 0.42	–
	DA	20.21 ± 5.44	16.12 ± 2.01	8.29 ± 1.83	11.16 ± 3.77	–

**Table 2:** The main pharmacokinetic parameters after oral administration of 25, 50, and 100 mg/kg for MOIG, and 1650 mg/kg for MO or intravenous administration of 25 mg/kg MOIG in female rats (*n* = 3, mean ± SD)

Parameters	Component	po-MOIG-25	po-MOIG-50	po-MOIG-100	po-MO-1650	iv-MOIG-25
AUC_0-t_ (μg·h/L)	MON	1988 ± 870	3083 ± 1184	2963 ± 397	1504 ± 244	19,098 ± 4740
	DA	3133 ± 1580	6572 ± 884	5727 ± 992	4254 ± 3301	8910 ± 2343
AUC_0-∞_ (μg·h/L)	MON	2040 ± 815	3178 ± 1290	2981 ± 400	1507 ± 243	19,129 ± 4743
	DA	3221 ± 1536	6661 ± 935	5785 ± 1024	4308 ± 3367	8945 ± 2349
AUMC_0-∞_ (μg·h/L)	MON	10,005 ± 3510	16,892 ± 14,358	8895 ± 1596	5589 ± 407	17,847 ± 2842
	DA	20,444 ± 6912	41,101 ± 9585	29,163 ± 7495	25,789 ± 23,049	9214 ± 715
MRT (h)	MON	5.28 ± 2.38	4.78 ± 2.64	2.99 ± 0.43	3.75 ± 0.34	0.98 ± 0.31
	DA	6.77 ± 1.35	6.12 ± 0.60	4.99 ± 0.44	5.66 ± 0.71	1.08 ± 0.31
t_1/2_ (h)	MON	3.61 ± 1.03	4.01 ± 2.35	2.08 ± 0.50	2.51 ± 0.34	1.76 ± 1.32
	DA	4.43 ± 1.12	3.66 ± 0.35	3.64 ± 0.43	3.38 ± 0.44	2.20 ± 1.86
T_max_ (h)	MON	0.94 ± 0.00	1.33 ± 0.29	0.92 ± 0.14	0.92 ± 0.14	0.03 ± 0.00
	DA	2.17 ± 0.76	1.83 ± 0.58	2.33 ± 0.58	2.08 ± 1.18	0.03 ± 0.00
V_d_ (L/g)	MON	0.08 ± 0.05	0.09 ± 0.04	0.10 ± 0.04	4.07 ± 1.16	0.003 ± 0.002
	DA	0.07 ± 0.06	0.04 ± 0.01	0.09 ± 0.01	2.50 ± 1.25	0.009 ± 0.006
CL (L/g·h)	MON	0.014 ± 0.005	0.018 ± 0.009	0.034 ± 0.05	1.114 ± 0.183	0.001 ± 0.000
	DA	0.010 ± 0.006	0.008 ± 0.001	0.018 ± 0.003	0.532 ± 0.287	0.003 ± 0.001
C_max_ (μg/L)	MON	475 ± 160	761 ± 184	899 ± 197	405 ± 102	25,719 ± 12,174
	DA	445 ± 218	863 ± 112	966 ± 171	584 ± 419	12,340 ± 5992
F (%)	MON	10.66 ± 4.26	8.31 ± 3.37	3.90 ± 0.52	3.94 ± 0.64	–
	DA	36.01 ± 17.17	37.23 ± 5.23	16.17 ± 2.86	24.08 ± 18.82	–

### Tissue distribution study

Tissue distribution of MON and DA was investigated in male and female rats at 0.5, 1, 2, 4, 8, 12 and 24 h after oral administration at a dose of 100 mg/kg MOIG. The level of MON and DA in tissues or organs including the small intestine, large intestine, stomach, spleen, liver, lung, kidney, heart, marrow, thymus, hypothalamus, testis, ovary and uterus was determined. As shown in Tables 3 and 4, MON and DA were widely distributed in all tissues examined after oral administration. MON and DA extensively distributed into the extra-vascular system of the animals. MON and DA levels are significantly reduced to an undetectable level in 12 h or 24 h after oral administration. In male rats, the highest concentration of MON and DA was observed in the intestine and stomach, followed by the spleen, heart, kidney, and testis at 1, 2, and 24 h after oral administration, while the highest concentration of MON and DA in female rats was found in hypothalamus, ovary, uterus, marrow, and liver at 0.5 and 1 h after oral administration. Apart from the intestine and stomach, the spleen and heart had a higher concentration of MON and DA in the male rats. The concentration of MON and DA in the liver, marrow and hypothalamus was higher in female rats than that in male rats.

**Table 3:** The concentrations (ng/mL) of MON and DA in various tissues after oral administration of 100 mg/kg MOIG in male rats (mean ± SD, *n* = 3)

Tissue/organs	Component	0.5 h	1 h	2 h	4 h	8 h	12 h	24 h
Small intestine	MON	162.8 ± 134.4	19,075.1 ± 16,724.9	3223.4 ± 3453.3	69.2 ± 114.2	24.6 ± 34.1	45.6 ± 78.9	0.1 ± 0.1
	DA	132.2 ± 101.9	13,371.3 ± 11,978.3	2053.3 ± 2478.3	495.0 ± 838.1	110.7 ± 152.2	124.9 ± 197.2	4.3 ± 6.2
Large intestine	MON	706.8 ± 659.0	924.9 ± 972.6	576.9 ± 380.8	66.1 ± 79.2	58.3 ± 29.9	18.6 ± 4.0	1.7 ± 3.0
	DA	2564.7 ± 3177.5	1928.8 ± 1640.6	3135.9 ± 2828.6	1310.0 ± 2008.0	994.9 ± 873.6	334.6 ± 69.8	26.0 ± 23.5
Stomach	MON	2888.7 ± 235.2	1052.1 ± 402.1	364.5 ± 141.9	11.7 ± 11.8	91.0 ± 21.5	12.1 ± 20.9	1.0 ± 1.7
	DA	2451.1 ± 638.1	1124.3 ± 210.2	435.1 ± 96.7	39.5 ± 21.6	84.1 ± 34.5	20.9 ± 34.5	3.2 ± 5.5
Spleen	MON	14.6 ± 9.8	75.2 ± 71.0	5434.0 ± 9376.5	66.6 ± 113.9	4.6 ± 7.9	0.2 ± 0.3	49.8 ± 86.2
	DA	14.1 ± 8.7	58.9 ± 49.4	3563.4 ± 6127.5	9.5 ± 1.9	6.6 ± 6.1	3.2 ± 4.6	29.6 ± 51.2
Testis	MON	18.4 ± 3.8	16.3 ± 15.3	56.6 ± 11.3	188.4 ± 304.1	304.2 ± 513.5	0.0 ± 0.0	0.0 ± 0.0
	DA	17.1 ± 4.9	79.1 ± 67.0	69.1 ± 11.2	24.8 ± 5.6	221.0 ± 358.6	0.0 ± 0.0	0.8 ± 1.3
Heart	MON	24.3 ± 10.6	545.5 ± 908.9	8.9 ± 1.9	0.0 ± 0.0	0.2 ± 0.3	0.0 ± 0.0	269.2 ± 466.2
	DA	22.7 ± 7.4	533.2 ± 856.8	28.9 ± 0.8	10.0 ± 1.9	2.2 ± 1.9	0.0 ± 0.0	214.3 ± 371.2
Kidney	MON	48.6 ± 19.5	147.8 ± 109.9	30.7 ± 4.3	17.0 ± 4.3	3.5 ± 1.7	4.2 ± 7.2	0.0 ± 0.0
	DA	91.7 ± 15.9	303.2 ± 162.0	220.2 ± 19.4	202.2 ± 40.2	66.1 ± 6.5	31.5 ± 18.8	2.5 ± 4.4
Marrow	MON	82.8 ± 46.8	24.2 ± 23.6	10.1 ± 2.9	5.8 ± 6.6	1.2 ± 2.0	0.0 ± 0.0	0.0 ± 0.0
	DA	102.7 ± 90.0	37.8 ± 21.0	24.8 ± 5.1	11.4 ± 3.3	5.6 ± 7.0	0.0 ± 0.0	0.0 ± 0.0
Liver	MON	15.6 ± 7.0	26.5 ± 7.9	20.3 ± 5.2	10.3 ± 3.9	12.6 ± 9.3	20.4 ± 35.3	0.0 ± 0.0
	DA	17.5 ± 2.4	46.9 ± 15.9	52.0 ± 7.6	35.4 ± 7.7	20.9 ± 5.4	22.9 ± 31.9	0.0 ± 0.0
Lung	MON	58.9 ± 29.3	28.7 ± 23.1	34.7 ± 13.9	9.5 ± 6.3	2.0 ± 0.3	64.3 ± 96.8	0.0 ± 0.0
	DA	47.5 ± 23.8	47.1 ± 8.6	48.5 ± 10.5	23.9 ± 2.5	6.4 ± 0.7	72.4 ± 116.7	3.9 ± 6.7
Thymus	MON	16.8 ± 11.5	11.6 ± 3.8	13.0 ± 2.8	13.0 ± 11.1	6.4 ± 1.3	0.0 ± 0.0	0.0 ± 0.0
	DA	22.7 ± 14.6	23.0 ± 3.5	29.7 ± 5.0	10.1 ± 1.9	4.5 ± 3.2	0.8 ± 1.4	1.5 ± 2.0
Hypothalamus	MON	152.5 ± 76.3	110.3 ± 58.0	122.7 ± 137.7	27.7 ± 39.7	16.4 ± 14.2	0.0 ± 0.0	0.0 ± 0.0
	DA	101.0 ± 19.9	70.9 ± 27.6	74.7 ± 88.3	32.5 ± 29.7	17.1 ± 14.9	4.8 ± 8.3	2.6 ± 4.6

**Table 4:** The concentrations (ng/mL) of MON and DA in various tissues after oral administration of 100 mg/kg MOIG in female rats (mean ± SD, *n* = 3)

Tissue/organs	Component	0.5 h	1 h	2 h	4 h	8 h	12 h	24 h
Small intestine	MON	244.3 ± 392.8	19,223.7 ± 9918.4	11,552.4 ± 18,012.3	5751.1 ± 4091.9	26.1 ± 20.4	8.3 ± 14.4	1.9 ± 3.2
	DA	153.0 ± 232.2	11,548.8 ± 3298.3	7897.3 ± 11,589.7	6001.6 ± 2129.6	28.3 ± 10.9	198.7 ± 310.3	3.0 ± 5.2
Large intestine	MON	966.5 ± 1501.4	1089.2 ± 411.5	2949.1 ± 4063.5	215.6 ± 137.4	27.9 ± 5.8	17.4 ± 22.5	0.0 ± 0.0
	DA	1408.8 ± 2274.2	2026.4 ± 1904.6	5383.1 ± 4417.7	5200.8 ± 4470.2	246.3 ± 189.6	374.1 ± 418.7	0.4 ± 0.3
Stomach	MON	1913.2 ± 1817.5	3098.1 ± 3293.7	1226.6 ± 1009.2	39.1 ± 22.2	81.3 ± 25.5	0.0 ± 0.00	0.0 ± 0.0
	DA	1526.7 ± 1436.1	3015.9 ± 3232.2	1328.7 ± 1031.1	83.0 ± 33.6	73.6 ± 22.8	1.0 ± 0.9	0.0 ± 0.0
Spleed	MON	25.0 ± 16.2	102.8 ± 120.3	28.5 ± 21.9	7.7 ± 7.1	14.9 ± 22.5	0.0 ± 0.0	0.0 ± 0.0
	DA	29.4 ± 15.7	83.9 ± 82.6	43.6 ± 31.6	19.9 ± 11.5	16.5 ± 17.7	0.0 ± 0.0	0.0 ± 0.0
Ovary	MON	374.7 ± 318.2	2112.7 ± 2497.3	504.0 ± 470.3	138.4 ± 105.5	45.5 ± 15.6	6.5 ± 11.3	1.7 ± 2.9
	DA	241.9 ± 185.0	854.1 ± 859.3	382.3 ± 298.2	110.4 ± 101.8	33.8 ± 9.5	5.7 ± 9.8	0.7 ± 1.3
Uterus	MON	331.7 ± 229.3	2214.1 ± 2264.0	601.3 ± 664.6	269.9 ± 217.3	97.2 ± 21.0	0.0 ± 0.0	3.0 ± 5.0
	DA	194.0 ± 122.7	1069.7 ± 908.8	426.3 ± 422.7	256.6 ± 340.3	68.4 ± 14.9	1.8 ± 2.1	3.6 ± 3.5
Heart	MON	44.3 ± 18.2	79.0 ± 91.0	21.5 ± 3.6	5.8 ± 5.7	3.7 ± 6.5	0.0 ± 0.0	0.0 ± 0.0
	DA	45.8 ± 19.1	86.1 ± 81.8	42.1 ± 7.7	17.6 ± 4.9	6.3 ± 5.6	0.0 ± 0.0	0.0 ± 0.0
Kidney	MON	146.6 ± 133.3	139.8 ± 98.6	68.3 ± 25.4	48.8 ± 27.5	14.1 ± 10.3	0.0 ± 0.0	8.1 ± 14.0
	DA	1832.9 ± 2990.7	218.6 ± 114.6	264.8 ± 33.7	244.0 ± 25.8	109.0 ± 22.5	44.6 ± 2.5	0.9 ± 1.4
Marrow	MON	583.1 ± 978.7	257.0 ± 226.1	48.4 ± 60.3	10.7 ± 14.8	0.1 ± 0.2	0.0 ± 0.0	0.0 ± 0.0
	DA	446.2 ± 744.7	209.9 ± 183.6	66.1 ± 51.3	27.6 ± 29.1	3.8 ± 1.9	0.0 ± 0.0	0.0 ± 0.0
Liver	MON	729.3 ± 993.8	169.0 ± 129.8	66.4 ± 23.4	13.9 ± 4.6	33.0 ± 36.6	0.0 ± 0.0	0.0 ± 0.0
	DA	536.2 ± 725.7	142.0 ± 83.4	87.2 ± 13.9	42.2 ± 12.1	36.3 ± 14.4	3.1 ± 3.0	0.0 ± 0.0
Lung	MON	72.2 ± 53.3	398.3 ± 491.2	50.6 ± 33.7	244.8 ± 407.7	26.7 ± 25.6	47.6 ± 82.5	0.0 ± 0.0
	DA	56.5 ± 29.8	222.3 ± 257.4	72.1 ± 28.2	180.7 ± 267.3	23.8 ± 16.9	30.2 ± 52.3	0.0 ± 0.0
Thymus	MON	22.3 ± 9.2	140.0 ± 144.2	32.6 ± 36.1	1.8 ± 1.1	22.8 ± 6.3	0.0 ± 0.00	0.0 ± 0.0
	DA	26.50 ± 7.7	136.05 ± 128.2	57.3 ± 45.6	14.1 ± 3.4	30.6 ± 8.6	0.0 ± 0.0	0.0 ± 0.0
Hypothalamus	MON	2209.3 ± 3684.2	504.1 ± 333.9	226.2 ± 264.4	41.1 ± 43.2	72.3 ± 95.2	0.0 ± 0.0	9.7 ± 16.9
	DA	1754.0 ± 2942.7	287.1 ± 176.9	145.8 ± 144.6	47.2 ± 9.2	67.7 ± 77.7	0.0 ± 0.0	21.0 ± 36.4

## Discussion

**Fig. 4:**
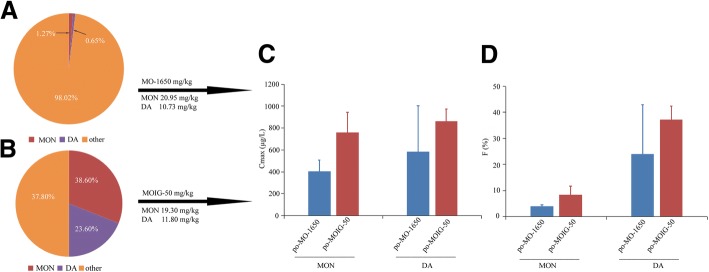
The pie diagram of actual values of MON and DA in the MO (**a**) and MOIG (**b**). After oral administration of the 50 mg/kg MOIG and 1650 mg/kg MO extract, comparing the C_max_ (**c**) and absolute bioavailability (**d**) of MON and DA in female rats plasma

It was found in the present study that the same dosage of MON and DA produced significantly different pharmacokinetic parameters in the treatment of po-MOIG-50 and po-MO-1650. Their comparisons were shown in Fig. 4. The AUC_0-t,_ AUC_0-∞_, C_max_ and absolute bioavailability of MON and DA in the treatment of po-MO-1650 were lower than those in the treatment of po-MOIG-50; the V_d_ and CL of MON and DA in the treatment of po-MO-1650 were by far higher than those in the treatment of po-MOIG-50. These results suggest that MON and DA were eliminated more quickly and distributed in the tissue under the condition of coexistence of multicomponents in MO-1650.

It was reported that gender was a potential factor affecting drug pharmacokinetics [18], including the absorption process, distribution and bioavailability. The present study showed that the pharmacokinetic parameters for male and female rats were significantly different. The AUC_0-t_, C_max_ and bioavailability of MON and DA in female rats were higher than those in male rats, but V_d_ and CL of MON and DA in female rats were lower than those in male rats, indicating that MON and DA were cleared more slowly in female rats than those in male rats.

MON and DA are isomers. Some studies [18, 19] demonstrated that MON and DA were relatively stable in the MO root under normal conditions, while MON may convert to DA in acidic conditions. This may be the reason for the lower bioavailability of MON than DA. In addition, the concentration-time curve of DA showed an obvious double-peak phenomenon. This might be caused by different absorption capacities in different regions of the gut or enterohepatic circulation [20], the conversion of MON or its derivatives into DA through the hydration in gastric acid conditions [17, 18], the pharmacological effect of the MON and DA [21], and gastric emptying-limited absorption [22].

However, we found that MON and DA also distributed in the hypothalamus, implying that they could pass through the blood-brain barrier. The samples collected at 12 h after administration indicated that MON and DA were gradually cleared and no accumulation was observed in the tissues. The compounds absorbed through the blood were transported to the target tissue, and then the unbound portion of the drug exerted the pharmacological effect. MON and DA distributed in the heart, liver, spleen, lung, kidney, especially in the thymus and bone marrow. Thence, we surmised that MON and DA may be able to exert their pharmacological effects in these target organs. MO strengths kidney-*yang* and improves spermatogenesis and the reproductive capacity [23, 24], which is consistent with our finding that MON and DA distributed in the testes at a high concentration. MO and MON showed an obvious anti-osteoporosis effect [10, 11], which is consistent with our finding that MON and DA were also observed in the ovary, uterus and bone marrow at a high concentration. The high concentration of MON and DA in the intestine may be related to their therapeutic effect against colitis [12]. The highest concentration of MON and DA in the hypothalamus was observed at 0.5 h, indicating that MON and DA could directly cross the blood-brain barrier and exert their potential pharmacological action on the hypothalamus-gonad system. In addition, the results of tissue distribution of MON and DA maybe implied some new therapeutic areas of MO. The distribution of MON and DA from MO in the stomach, intestine and lung, combined with its anti-inflammatory effects, maybe hint that MO can be used to prevent and treat inflammatory disease in these organs, such as gastritis, pneumonia and colon cancer.

Regarding the toxicity of the MO, there is no literature to report the adverse effect of MO at a normal dose in clinics. The acute toxicity test indicated that MO at a cumulative dose of 250 g/kg/day did not lead to death of mice in 3 days [25]. Some investigation showed that MO extracts had no mutagenic or genotoxic effect on *Escherichia coli* PQ37DNA [26]. Our experiments indicated that MOIG at dose of 22.5 g/kg did not cause any death of mice. These evidence, together with their significant pharmacological properties, implied that iridoid glycosides, such as monotropein and deacetyl asperulosidic acid from Morinda officinalis root in rats, had potential for the use in medication, especially for inflammatory disease.

## Conclusion

The two major IGs (MON and DA) from the MO root were simultaneously determined by a simple, rapid and sensitive UHPLC-MS/MS method. This method was also used in the study of pharmacokinetics and tissue distribution after oral administration of 25, 50, and 100 mg/kg MOIGs and 1650 mg/kg MO extract. This is the first report on the pharmacokinetic and tissue distribution of MON and DA after oral administration of the MO extract. We also found that MON and DA exhibited a significant gender difference in terms of the pharmacokinetic parameters. In addition, the absolute bioavailability of MON and DA also showed a significant gender difference. The results of tissue distribution in male and female rats indicated that MON and DA from the MO root mainly distributed in the intestine and stomach after oral administration, followed by the spleen, hypothalamus, and gonad. These findings may shed new lights on the biological behavior of MOIGs in vivo*,* help explain some of their pharmacological actions, and provide experimental clues for rational clinical use of these IGs extracted from the MO root.

## Additional files

Additional file 1: **Table S1**. Standard curves, linear ranges, correlation coefficients and lower limit of quantification of MON and DA in biological samples. (DOC 68 kb)

Additional file 2: **Table S2**. Intra-day and inter-day accuracy and precision of analytes in rats blank samples. (DOC 19 kb)

Additional file 3: **Table S3**. Extraction recovery and matrix effect of MON, DA and IS in rat plasma and tissue homogenates (*n* = 6, mean ± SD). (DOC 83 kb)

Additional file 4: **Table S4**. Dilution integrity experiments of MON and DA (*n* = 5, mean ± SD). (DOC 19 kb)

Additional file 5: **Table S5**. Stability of MON and DA in blank plasma samples (*n* = 5). (DOC 19 kb)

## Abbreviations

ACE: Acetaminophen
AJS: Agilent jet stream technology
AUC_0-∞_: Area under the plasma concentration-time curve from zero to time infinity
AUC_0-t_: Area under the plasma concentration-time curve
CL: Body clearance
C_max_: Peak concentration
DA: Deacetyl asperulosidic acid
DSS: Dextran sulfate sodium
EMA: European Medicines Agency
ESI: Electrospray source interface
FDA: Food and Drug Administration
F_oral_: Absolute oral bioavailability
IGs: Iridoid glycosides
IL-1β: Interleukin-1-beta
IS: Internal standard
LLOQ: Lower limit of quantification
ME: Matrix effect
MO: *Morinda officinalis*
MOIGs: *Morinda officinalis* iridoid glycosides
MON: Monotropein
MRM: Multiple reaction monitoring
MRT: Mean residence time
QC: Quality control
t_1/2_: Half-life
T_max_: Peak time
UHPLC-MS: Ultra high performance liquid chromatography-tandem mass spectrometry
ULOQ: Upper limit of quantification
V_d_: Apparent volume of body distribution
