# Supplementary material for: Pharmacokinetics and tissue distribution of monotropein and deacetyl asperulosidic acid after oral administration of extracts from Morinda officinalis root in rats
Source: BMC Complement Altern Med. 2018 Oct 24;18:288. doi: 10.1186/s12906-018-2351-1 (PMC6201592; doi:10.1186/s12906-018-2351-1)
Supplement: Supplementary file 1 — Table S1. Standard curves, linear ranges, correlation coefficients and lower limit of quantification of MON and DA in biological samples. (DOC 68 kb) [file 12906_2018_2351_MOESM1_ESM.doc]

**Table S1** Standard curves, linear ranges, correlation coefficients and lower limit of quantification of MON and DA in biological samples.

| Bio-samples | Compound | Standard curves | Linear ranges (ng/mL) | correlation coefficients (R) | LLOQ (ng/mL) |
| --- | --- | --- | --- | --- | --- |
| Plasma | MON | *y* = 1.88 *x* + 9.28 | 2-5000 | 0.9939 | 2 |
|  | DA | *y* = 5.63 *x* + 6.97 | 2-5000 | 0.9932 | 2 |
| Stomach | MON | *y* = 7.01 × 10^-4^ *x* + 0.01 × 10^-3^ | 2-5000 | 0.9971 | 2 |
|  | DA | *y* = 8.52 × 10^-5^ *x* + 5.45 × 10^-4^ | 2-5000 | 0.9986 | 2 |
| Spleen | MON | *y* = 1.18 × 10^-3^ *x* + 4.33 × 10^-2^ | 2-5000 | 0.9966 | 2 |
|  | DA | *y* = 3.40 × 10^-4^ *x* + 2.65 × 10^-3^ | 2-5000 | 0.9966 | 2 |
| Testis | MON | *y* = 5.56 × 10^-4^ *x* + 1.55 × 10^-3^ | 2-5000 | 0.9975 | 2 |
|  | DA | *y* = 7.06 × 10^-4^ *x* + 1.41 × 10^-3^ | 2-5000 | 0.9961 | 2 |
| Heart | MON | *y* = 2.67 × 10^-4^ *x* + 1.12 × 10^-3^ | 2-5000 | 0.9951 | 2 |
|  | DA | *y* = 2.65 × 10^-4^ *x* + 8.35 × 10^-4^ | 2-5000 | 0.9933 | 2 |
| Ovary | MON | *y* = 1.08 × 10^-3^ *x* + 1.25 × 10^-2^ | 2-5000 | 0.9930 | 2 |
|  | DA | *y* = 1.79 × 10^-4^ *x* + 1.16 × 10^-3^ | 2-5000 | 0.9949 | 2 |
| Uterus | MON | *y* = 1.30 × 10^-3^ *x* + 1.41 × 10^-2^ | 2-5000 | 0.9902 | 2 |
|  | DA | *y* = 3.19 × 10^-4^ *x* + 2.24 × 10^-3^ | 2-5000 | 0.9960 | 2 |
| Kidney | MON | *y* = 5.28 × 10^-4^ *x* + 8.88 × 10^-4^ | 2-5000 | 0.9987 | 2 |
|  | DA | y = 2.98 × 10^-4^ *x* + 5.38 × 10^-4^ | 2-5000 | 0.9971 | 2 |
| Marrow | MON | y = 5.76 × 10^-4^ *x* + 2.88 × 10^-3^ | 2-5000 | 0.9907 | 2 |
|  | DA | y = 7.64 × 10^-4^ *x* + 2.36 × 10^-3^ | 2-5000 | 0.9911 | 2 |
| Liver | MON | *y* = 4.39 × 10^-4^ *x* + 4.86 × 10^-4^ | 2-5000 | 0.9881 | 2 |
|  | DA | y = 9.56 × 10^-5^ *x* + 2.22 × 10^-4^ | 2-5000 | 0.9900 | 2 |
| Lung | MON | y = 1.04 × 10^-3^ *x* + 2.56 × 10^-3^ | 2-5000 | 0.9928 | 2 |
|  | DA | y = 2.90 × 10^-4^ *x* + 2.46 × 10^-4^ | 2-5000 | 0.9954 | 2 |
| Thymus | MON | y = 2.95 × 10^-4^ *x* + 2.07 × 10^-3^ | 2-5000 | 0.9974 | 2 |
|  | DA | y = 4.90 × 10^-4^ *x* + 2.78 × 10^-3^ | 2-5000 | 0.9972 | 2 |
| Small intestine | MON | y = 7.13 × 10^-4^ *x* + 1.80 × 10^-3^ | 2-5000 | 0.9979 | 2 |
|  | DA | y = 4.06 × 10^-4^ *x* + 1.39 × 10^-3^ | 2-5000 | 0.9994 | 2 |
| Large intestine | MON | y = 7.21 × 10^-4^ *x* + 3.92 × 10^-3^ | 2-5000 | 0.9959 | 2 |
|  | DA | y = 3.59 × 10^-4^ *x* + 6.11 × 10^-4^ | 2-5000 | 0.9959 | 2 |
| Hypothalamus | MON | y = 1.63 × 10^-3^ *x* - 5.55 × 10^-4^ | 2-5000 | 0.9940 | 2 |
|  | DA | y = 8.42 × 10^-4^ *x* + 2.66 × 10^-4^ | 2-5000 | 0.9965 | 2 |
