# Supplementary material for: Pharmacokinetics and tissue distribution of monotropein and deacetyl asperulosidic acid after oral administration of extracts from Morinda officinalis root in rats
Source: BMC Complement Altern Med. 2018 Oct 24;18:288. doi: 10.1186/s12906-018-2351-1 (PMC6201592; doi:10.1186/s12906-018-2351-1)
Supplement: Supplementary file 2 — Table S2. Intra-day and inter-day accuracy and precision of analytes in rats blank samples. (DOC 19 kb) [file 12906_2018_2351_MOESM2_ESM.doc]

**Table S2** Intra-day and inter-day accuracy and precision of analytes in rats blank samples.

| Concentration  (ng/mL) | MON | | | | DA | | | | |
| --- | --- | --- | --- | --- | --- | --- | --- | --- | --- |
|  | Precision (RSD%) | | Accuracy (RE%) | | Precision (RSD%) | | Accuracy (RE%) | |  |
|  | Intra-day  (n=5) | Inter-day  (n=15) | Intra-day  (n=5) | Inter-day  (n=15) | Intra-day  (n=5) | Inter-day  (n=15) | Intra-day  (n=5) | Inter-day  (n=15) | |
| 5 | 9.26 | 7.48 | -9.14 | -8.95 | 5.75 | 3.83 | -5.41 | 7.99 | |
| 1000 | 0.81 | 1.12 | 4.28 | -1.17 | 2.39 | 2.39 | 0.46 | 8.09 | |
| 4000 | 0.79 | 1.55 | 2.16 | -3.51 | 5.19 | 5.01 | -7.72 | 1.86 | |
