# Supplementary material for: Pharmacokinetics and tissue distribution of monotropein and deacetyl asperulosidic acid after oral administration of extracts from Morinda officinalis root in rats
Source: BMC Complement Altern Med. 2018 Oct 24;18:288. doi: 10.1186/s12906-018-2351-1 (PMC6201592; doi:10.1186/s12906-018-2351-1)
Supplement: Supplementary file 3 — Table S3. Extraction recovery and matrix effect of MON, DA and IS in rat plasma and tissue homogenates (n = 6, mean ± SD). (DOC 83 kb) [file 12906_2018_2351_MOESM3_ESM.doc]

**Table S3 Extraction recovery and matrix effect of MON, DA and IS in rat plasma and tissue homogenates (n=6, mean±SD)**

| Bio-samples | Concentration (ng/mL) | MON | | DA | |
| --- | --- | --- | --- | --- | --- |
|  |  | Extraction recovery (%) | Matrix effects (%) | Extraction recovery (%) | Matrix effects (%) |
| Plasma | 5 | 63.95 ± 9.01 | 101.24 ± 6.48 | 90.00 ± 9.03 | 99.65 ± 7.17 |
|  | 1000 | 65.42 ± 1.30 | 93.29 ± 0.85 | 88.11 ± 2.45 | 85.87 ± 1.39 |
|  | 4000 | 62.44 ± 2.04 | 99.98 ± 1.90 | 90.66 ± 2.44 | 92.90 ± 1.18 |
| Stomach | 5 | 95.34 ± 6.72 | 95.67 ± 10.01 | 92.06 ± 10.62 | 101.81 ± 11.42 |
|  | 1000 | 96.36 ± 1.23 | 65.54 ± 1.46 | 83.99 ± 4.87 | 84.28 ± 4.00 |
|  | 4000 | 100.01 ± 1.64 | 61.31 ± 0.82 | 77.51 ± 3.02 | 92.13 ± 4.00 |
| Spleen | 5 | 92.87 ± 5.59 | 95.23 ± 9.02 | 100.32 ± 13.00 | 96.73 ± 12.37 |
|  | 1000 | 97.41 ± 0.80 | 88.18 ± 0.88 | 100.80 ± 1.56 | 99.42 ± 3.33 |
|  | 4000 | 92.63 ± 0.39 | 91.22 ± 0.96 | 98.63 ± 0.90 | 105.19 ± 1.81 |
| Testis | 5 | 62.98 ± 12.30 | 65.98 ± 10.12 | 83.55 ± 13.51 | 97.12 ± 8.69 |
|  | 1000 | 53.19 ± 1.58 | 51.08 ± 1.19 | 87.02 ± 2.74 | 107.28 ± 2.05 |
|  | 4000 | 54.47 ± 0.69 | 43.77 ± 0.23 | 84.81 ± 2.09 | 102.09 ± 2.14 |
| Heart | 5 | 72.18 ± 4.75 | 21.95 ± 1.24 | 92.06 ± 14.09 | 69.54 ± 10.43 |
|  | 1000 | 70.63 ± 2.38 | 23.54 ± 0.29 | 94.72 ± 2.33 | 76.10 ± 2.08 |
|  | 4000 | 71.39 ± 0.99 | 23.52 ± 0.27 | 94.17 ± 1.05 | 79.43 ± 0.93 |
| Ovary | 5 | 95.14 ± 9.06 | 79.22 ± 10.91 | 91.65 ± 12.21 | 97.01 ± 13.23 |
|  | 1000 | 89.18 ± 1.02 | 76.73 ± 0.92 | 96.82 ± 2.92 | 95.74 ± 3.17 |
|  | 4000 | 98.02 ± 1.41 | 78.20 ± 0.76 | 108.58 ± 0.70 | 98.95 ± 1.11 |
| Uterus | 5 | 76.86 ± 8.61 | 106.63 ± 3.34 | 84.59 ± 17.50 | 90.75 ± 14.25 |
|  | 1000 | 57.51 ± 3.11 | 97.11 ± 0.58 | 71.71 ± 2.58 | 102.46 ± 1.07 |
|  | 4000 | 61.13 ± 1.12 | 102.61 ± 1.52 | 72.56 ± 2.79 | 112.65 ± 4.32 |
| Kidney | 5 | 80.88 ± 5.50 | 78.46 ± 8.17 | 99.93 ± 5.36 | 42.78 ± 0.39 |
|  | 1000 | 90.13 ± 2.20 | 37.61 ± 1.44 | 69.88 ± 1.48 | 96.61 ± 2.55 |
|  | 4000 | 99.24 ± 3.06 | 16.38 ± 0.69 | 78.92 ± 1.95 | 107.62 ± 8.56 |
| Marrow | 5 | 54.81 ± 12.25 | 64.04 ± 9.30 | 85.46 ± 14.23 | 135.48 ± 13.80 |
|  | 1000 | 51.56 ± 0.64 | 54.90 ± 0.73 | 81.44 ± 1.68 | 130.00 ± 1.65 |
|  | 4000 | 60.61 ± 0.76 | 50.51 ± 0.46 | 88.39 ± 1.54 | 123.50 ± 3.26 |
| Liver | 5 | 54.42 ± 6.16 | 67.64 ± 9.36 | 56.92 ± 11.26 | 50.80 ± 6.42 |
|  | 1000 | 60.94 ± 1.25 | 61.15 ± 1.57 | 60.94 ± 3.35 | 44.69 ± 1.79 |
|  | 4000 | 59.34 ± 0.97 | 62.87 ± 1.04 | 56.78 ± 1.22 | 47.79 ± 0.99 |
| Lung | 5 | 101.63 ± 8.05 | 106.15 ± 7.64 | 98.35 ± 13.04 | 99.34 ± 12.57 |
|  | 1000 | 88.00 ± 0.69 | 95.11 ± 0.94 | 94.12 ± 1.54 | 103.19 ± 2.07 |
|  | 4000 | 92.76 ± 0.55 | 92.73 ± 0.94 | 99.56 ± 0.91 | 102.07 ± 0.99 |
| Thymus | 5 | 67.09 ± 5.56 | 32.84 ± 3.90 | 81.75 ± 10.79 | 55.86 ± 6.65 |
|  | 1000 | 63.42 ± 5.04 | 11.25 ± 0.44 | 90.35 ± 0.71 | 50.77 ± 0.74 |
|  | 4000 | 64.62 ± 6.92 | 9.48 ± 0.20 | 82.89 ± 2.14 | 47.01 ± 0.65 |
| Small intestine | 5 | 100.01 ± 14.18 | 53.66 ± 6.60 | 74.59 ± 11.70 | 99.20 ± 11.89 |
|  | 1000 | 94.25 ± 1.65 | 49.74 ± 0.89 | 61.97 ± 0.85 | 100.88 ± 2.00 |
|  | 4000 | 94.41 ± 1.50 | 42.90 ± 0.97 | 60.48 ± 0.92 | 88.78 ± 1.94 |
| Large intestine | 5 | 97.82 ± 12.62 | 77.61 ± 6.15 | 103.73 ± 7.98 | 79.09 ± 9.22 |
|  | 1000 | 111.85 ± 1.03 | 51.33 ± 0.56 | 98.88 ± 1.45 | 79.89 ± 1.18 |
|  | 4000 | 101.01 ± 3.00 | 49.37 ± 0.42 | 89.28 ± 2.10 | 79.56 ± 0.93 |
| Hypothalamus | 5 | 74.53 ± 5.31 | 62.31 ± 4.41 | 94.40 ± 6.79 | 109.35 ± 7.72 |
|  | 1000 | 70.37 ± 1.20 | 55.17 ± 0.92 | 94.17 ± 1.29 | 108.20 ± 2.31 |
|  | 4000 | 68.74 ± 1.05 | 48.92 ± 0.69 | 92.22 ± 0.79 | 99.13 ± 1.79 |
| IS | 10 | 107.42 ± 4.11 | 109.26 ± 3.97 |  |  |
