# Supplementary material for: Pharmacokinetics and tissue distribution of monotropein and deacetyl asperulosidic acid after oral administration of extracts from Morinda officinalis root in rats
Source: BMC Complement Altern Med. 2018 Oct 24;18:288. doi: 10.1186/s12906-018-2351-1 (PMC6201592; doi:10.1186/s12906-018-2351-1)
Supplement: Supplementary file 4 — Table S4. Dilution integrity experiments of MON and DA (n = 5, mean ± SD). (DOC 19 kb) [file 12906_2018_2351_MOESM4_ESM.doc]

**Table S4** Dilution integrity experiments of MON and DA (n=5, mean ± SD)

| compound | Pre-diluted concentration (ng/mL) | Diluted measured concentration (ng/mL) | Precision  (RSD%) | Accuracy  (RE%) |
| --- | --- | --- | --- | --- |
| MON | 10000 | 1011.58 ± 82.26 | 8.13 | 1.93 |
|  | 20000 | 1834.71 ± 124.38 | 6.78 | -13.77 |
|  | 40000 | 3950.30 ± 328.37 | 8.31 | -2.07 |
| DA | 10000 | 1041.51 ± 98.89 | 9.49 | 6.92 |
|  | 20000 | 1889.62 ± 71.77 | 3.79 | -9.20 |
|  | 40000 | 3925.10 ± 330.82 | 8.43 | -3.12 |
