# Supplementary material for: Pharmacokinetics and tissue distribution of monotropein and deacetyl asperulosidic acid after oral administration of extracts from Morinda officinalis root in rats
Source: BMC Complement Altern Med. 2018 Oct 24;18:288. doi: 10.1186/s12906-018-2351-1 (PMC6201592; doi:10.1186/s12906-018-2351-1)
Supplement: Supplementary file 5 — Table S5. Stability of MON and DA in blank plasma samples (n = 5). (DOC 19 kb) [file 12906_2018_2351_MOESM5_ESM.doc]

**Table S5** Stability of MON and DA in blank plasma samples (n = 5).

| Concentration (ng/mL) | Stability (RE%) | | | | | | | | |
| --- | --- | --- | --- | --- | --- | --- | --- | --- | --- |
|  | Room temperature  (6 h, 25℃) | | Auto-sampler  (24 h, 4℃) | | Three freeze/thaw cycles | | Long term  (30 day, -80℃) | |  |
|  | MON | DA | MON | DA | MON | DA | MON | DA | |
| 5 | 5.97 | 4.84 | 7.94 | -3.84 | 3.80 | -6.86 | 2.08 | 14.08 | |
| 1000 | -7.85 | -6.56 | -8.61 | -7.00 | -5.35 | -6.54 | -10.38 | -9.58 | |
| 4000 | -3.75 | -6.06 | -3.50 | -6.08 | -4.80 | 5.67 | -5.11 | -8.09 | |
